# Philadelphia Hospital

**Published:** 1839-04-13

**Authors:** 


### CLINICAL LECTURE.

### PHILADELPHIA HOSPITAL.

ON PNEUMONIA—(CONTINUED.)

As an illustration of pneumonia, complicated with disease of the brain, I cannot do better than read to you the case of a young negro, of a slender frame, whom you have several times seen. It is an instance of arachnitis supervening towards the termination of the pneumonia, and therefore strictly secondary. You must have been already surprised at the very insidious nature of the disease, and the slight symptoms of the cerebral inflammation.

*Pneumonia with Arachnitis—Fatal Case.*

Charles Abriel, aged 22, entered the hospital January 8, 1839. He has recently been travelling through the country sweeping chimneys. Was taken ill on Friday the 3d; quite well previously; on that day was skating, after his return from a journey. On the night of the 3d to 4th, was seized with cough and spitting of blood, (about two ounces,) hæmoptysis not repeated; cough has continued ever since. The pain began the same night on the right side, near the nipple; prefers lying on the left side; chills irregularly through the day, but he is not conscious of sweating. Vomited only once while on his way to the hospital. Walked from the city in an hour, a distance of nearly two miles.

No treatment, drank no spirits.

*8th.*—Admitted during the morning visit; much exhausted; dyspnœa great. Pulse rather full; cough; bronchial respiration at anterior part of right side, from clavicle nearly to the base, with abundant crepitus in coughing; posteriorly on same side respiration rude in upper lobe, with distant crepitus; inferiorly respiration vesicular, nearly natural.

℞. Rad. Senega, ℥i.
Rad. Sanguinaria, ʒij.
in aq. Oj.—℥ij. every two hours.

In the evening had eight scarified cups applied to the anterior part of chest, on both sides, to take ℥viij. of blood; was neither puked nor purged by the infusion. Sweating profuse, particularly after the cupping in the evening.

*9th.*—Prostration considerable; decubitus on right side; dullness of intellect, but no lower nor delirium; nostrils dilating widely; eyes slightly injected; skin warm, a little moist; pulse one hundred and twenty-four, much fuller than yesterday, and slightly resisting; respiration fifty-six, noisy; expectoration very small, tenacious and whitish; tongue very red, rather smooth; no pain about abdomen; two stools; liver distended, passing two or three inches beyond rib; no tenderness, no nausea; great thirst. Percussion posteriorly flat at upper two-fifths of the right side; moderately sonorous inferiorly; dull at root of the left lung; respiration on right side at upper two-fifths intensely bronchial; very fine crepitus on coughing; crepitus throughout axilla and in lower lobe; rude respiration at middle of left lung, elsewhere respiration vesicular; bronchophony corresponds accurately with bronchial respiration. Anteriorly right side, bronchial respiration at summit; crepitus inferiorly; percussion dull; sounds of heart very feeble, impulse slight; dullness on percussion extends half an inch beyond nipple. Venesection pro re nata. Sinapism to lower extremities. Repeat the infusion every hour, until nausea.

*In evening.*—Pulse had again risen; some sub-crepitus was continued with the bronchial respiration, which was intense; was bled in the morning until he became faint, about ℥xv.; cupped to right side, No. iv. ℥vi. taken—afterwards was easy and went to sleep.

*10th.*—Took infusions of sanguinaria and senega every hour; was purged freely six times in four hours. In the afternoon diminished the dose to two ounces in every two hours. Expectoration has been very small in quantity; skin moist; no nausea occurred this morning, and no vomiting; cough a little looser; respiration nearly fifty in the minute. Continued infusion every two hours

*11th.*—Is still purged, three times in last four hours; three brown and very fœtid stools; no nausea; appetite returning for first time; sweating moderate; cough much more loose and frequent; expectoration, ℥i. to ℥iss. in twenty-four hours, thick, opaque, like half concrete albumen; dullness less marked; answers very readily; no cephalalgia; keeps well; dyspnœa; dilatation of nostrils less; pulse one hundred and ten, softer, and regular, but still a little resisting; respiration irregular; posteriorly on right side bronchial; respiration less intense in upper half; abundant crepitus, a little loose in lower half; tubal respiration in axilla, with no crepitus, but a little pleuritic creaking; anteriorly respiration vesicular and noisy; percussion flat at part where the bronchial respiration is heard. Discontinue infusion.

℞. Ipecac., gr. i. }

Calomel, gr. i. } q. 4 h.

Opii. gr. $\frac{1}{6}$ }

*11th.*—6 P. M. Bowels open twice since morning visit; slight diaphoresis; no nausea; no cephalalgia; pulse seventy-eight, soft and irregular; respiration forty-eight, more noisy than in morning; cough slight, and expectoration still of a dark reddish colour and glutinous consistence. Continue ipecac., &c.

*12th.*—Less dull, still disposed to sleep; bronchitis in affected side; no pain; cough more loose; expectoration yellowish, a little puriform; less injection of eyes; sputa moist, still covered with glutinous liquid; skin moist, moderately warm; pulse ninety-two, soft and regular; respiration twenty-eight; five stools since evening visit; *asks for food;* bronchial respiration continues to same extent and degree as yesterday. In axilla crepitus larger and looser. Continue treatment.

*12th.*—6 P. M. Bowels open four times since last visit. Slight diaphoresis at present; pulse one hundred and twenty, full and irregular; respiration, forty-eight, noisy; coughs more than in the morning; expectoration still slight, not so dark, and more frothy than in the morning.

℞.—Scarified cups, No. viii to posterior lower half of chest. Continue ipecacuanha, &c.

*13th*—Bowels open three times since last visit. Tongue red and coated with a whitish fur in patches; skin warm and moist; pulse, ninety, soft, but irregular; respiration, thirty-six, not so noisy as last night; eyes still injected; nostrils somewhat dilated; cough and expectoration slight.

℞.—Continue ipecacuanha.

13*th.*—6 P. M. Bowels open once since last visit. Tongue, red and dry; skin dry and hot; pulse, eighty-six, full and regular; respiration, thirty-two.

℞.—Continue treatment.

14*th.*—Somnolence; sputa moist, covered with a glutinous paste, like coat; no cephalalgia; intelligence clear; three stools in fifteen hours; appetite better; thirst less; no nausea; cough less violent and looser; expectoration mucous, nearly yellowish, not viscid; lies still on right side; sputa moist in the night; skin quite cool; pulse 104, feeble; respiration twenty-four, regular; tubal respiration on right side posteriorly continues in the upper two-fifths; in lower three-fifths vesicular, but feeble, with some crepitus; bronchial in axilla anteriorly; respiration rude, but vesicular.

℞.—Continue ipecacuanha. Mutton broth.

14*th.*—P. M. Continues about same as regards cough, expectoration, &c., as in the morning; pulse one hundred and four, full; respiration, thirty-two, regular and easy.

15*th.*—Bowels open three times since last visit; appetite improved; no pain, no cephalalgia; gums slightly affected; pulse one hundred and six, regular, and full; respiration, thirty-two, regular.

℞.—Continue ipecacuanha, every six hours.

On the 16th the countenance was more lively, the dyspnœa had almost ceased; cough looser; expectoration still characteristic, viscid, in part transparent, in part mucopurulent; respiration noisy, thirty; pulse eighty-two, irregular and soft; skin moist; tongue nearly clean, smooth and reddish; appetite returning; three stools in twenty-four hours. The respiration is becoming more vesicular at the root and lower lobe of the right lung; elsewhere there is but little change. Continue treatment.

On the 17th, the pulse was one hundred and two, and irregular; respiration forty-two. In other respects symptoms as before.

In the evening his pulse became more quick and corded; tongue dry and reddish, but the patient, instead of seeming more unwell, sat up half an hour, while the nurse was making his bed.

18*th.*—At noon he was in a state of profound stupor with injected conjunctivæ, and contracted pupils. Delirium had occurred in the morning; returns no answer to questions; skin moist and warm; pulse one hundred and twenty, quick and irritated, but rather feeble. Respiration thirty-five, a little stertorous; cough and expectoration have ceased since midnight; tongue very dry and red; respiration scarcely heard at the anterior part of the right side; vesicular at the left; sounds of heart so confused and rapid as to be scarcely distinguishable, but impulse very strong. Blister to occiput, ice to the head, sinapisms to extremities.

In the evening the pulse was one hundred and twenty; the delirium had continued throughout the day. Two discharges from the bowels.

19*th.*—Died at 12 o'clock.

*Autopsy.*

*Head.*—Arachnoid dry, especially on right side, adhering closely to convolutions; injection moderate, but it is formed of the smallest vessels, which seem to pass in abundance from cortical substance to pia mater; membranes throughout opaque, of a yellowish tint, much thickened, particularly near the tissue, where the thickening depends upon an effusion of a concrete yellowish substance. Lymph between arachnoid and pia mater; on left side the appearances are similar; the yellowish tint is most conspicuous near the middle and internal portions; the injection is decided, but much less between hemispheres; the cortical substance is brightly injected on both sides, particularly on right, which injection extends from finer vessels to medullary substance, giving it a yellow tint, also much marked on right side. The ventricles distended by three ounces of turbid serum, evidently mixed with purulent matter; the central portions are soft, but not diffluent; right ventricle, when fully opened, seemed smeared with a coating of lymph, which can be seized with the forceps, about the thickness of writing paper; in fact, however, it is thicker, softer, and not organized; same deposit extends to both ventricles, a little less abundant on left side than on right, it is most marked in both at their anterior extremity, over large extremity of optic thalami; the membrane itself is obviously thickened, and can be raised by forceps; it is easily detached, more injected, offering rather an orange tint, than distinct vessels.

*Base of brain.*—The prons varolii, medulla oblongata, are concealed completely by a coating of greenish lymph, filling up all the depressions, about the thickness of the derm, much thinner opposite the depressions, covers completely the origin of all the nerves, surrounds the medulla oblongata, but is less thick on posterior side, extends itself as a thin and incomplete covering over the inferior surface of the cerebellum, and follows the junction between it and cerebellum.

*Anteriorly*, it covers completely the commissure of the optic nerves, and is gradually lost about the middle of the olfactory, filling up on both sides the fissure of Sylvius, so that they cannot be separated without tearing the brain; rather more abundant on left than right side. The inferior lobes of the cerebrum very brightly injected without the effusion of lymph.

The consistence of the brain is generally good, as well as cerebellum, which is much paler than cerebrum.

*Spinal marrow.*—Membranes brightly injected exteriorly; the arachnoid, covering it, contains a coating of lymph, lining its inner surface; readily detached, soft; the spinal marrow itself is covered more or less completely with lymph, a portion of which is contained in the cavity of the arachnoid, another portion is combined with the membrane, rendering them thick, and strongly adherent to the medulla. Upper portion of spinal marrow softened, particularly at the posterior part, which extends more or less throughout the whole extent, (probably cadaveric.) Traces of lymph are to be found in the arachnoid, down as far as the cauda equina.

*Thorax.*—*Right lung*, adherent throughout with strong cellular adhesions; upper lobe solid to the feel, heavy, flat on percussion. Tissue presents a marbled aspect of gray and red, friable, a yellowish-red liquid exuding; granulations indistinct, (beginning of third stage;) at the summit the tissue is uniformly red, and more granulated; at the inferior angle there is still a portion permeable in part, but of a deep red colour. The middle lobe adheres closely to the upper, and is hepatized in irregular lobules. The lower lobe is much smaller than usual, while the upper is larger. Tissue of a deep red, contains some air, floats, but throughout is much less soft than natural, and is evidently at the commencement of hepatization, some portions being decidedly granulated. Bronchial tubes of the upper lobe, entirely red, thickened, containing a viscid, whitish liquid; in lower lobes the tubes are much injected, but containing less liquid than upper.

*Left lung* contains air throughout; grayish externally; upper lobe is entirely permeable, of a bright red colour, (almost arterial,) a little dry, and very crepitant, a reddish liquid is pressed from it, and contains but little air; lower lobe of a deep red colour, with abundance of reddish and very frothy serum; even at the root of the lung the tissue is still permeable. Bronchial tubes filled with reddish mucus; membrane red, particularly in larger divisions. This lung not adherent except at base.

*Pericardium* contains about three ounces of transparent serum; a few whitish patches on the pericardium, half organized, and capable of being detached with moderate effort in other portions of its extent; this membrane is a little opaque, but scarcely injected.

Large febrinous coagula in both sides of the heart, well organized with minute reddish lines, like newly formed vessels. The internal membrane is but slightly injected, and is moderately opaque. At the mitral valve is an ulceration a third of an inch in diameter, with everted edges; the rest of this valve is but little thickened with no vegetation or lymph. The semilunar valves are thin, and near the edges of two of them is an evident erosion, the membranes being gradually thickened, almost like tissue paper. The valves in the right side are not altered.

*Liver* much enlarged, especially in right lobe, which is greater than usual; tissue of an uniform red; where engorged with blood, a little softened; bile very small in quantity.

*Spleen* eight inches long, firm, of a dull purple colour.

*Kidneys* firm, of usual weight, membrane readily detached; in the right a lobule is yellow, infiltrated with a substance very closely resembling tuberculous matter.

*Aorta* pale.

*Stomach* contracted, contains a thick whitish mucus; membrane generally pale, rather thicker and softened, (appearances doubtful,) near the pylora, intense injections, in irregular ecchymosed spots.

*Small intestines.*—Mesenteric glands larger than usual, a few contains some grains of tuberculous matter.

*Mucous membrane* pale and firm; glands at the lower extremity are not enlarged nor injected.

*Large intestine* contains greenish pulpy fœces.

You have here a case of pneumonia, which, at first, presented but few unusual features; indeed, the patient was entering upon convalescence when the cerebral symptoms appeared. In the early period of the disease you may have remarked that the brain was slightly affected, but this was only to an insignificant degree, such as we often see in pneumonia. The cerebral symptoms then declined with the improvement of the patient, until they suddenly re-appeared, in an insidious and most obscure manner.

If you had witnessed only the pathological changes which were so evident after death, you would, in all probability, have affirmed that the ordinary symptoms of violent arachnitis had corresponded to the large deposit of pus in the membranes of the brain, as well as the partial destruction of the cerebral substance. But you will find that arachnitis, more especially of the base of the brain, and of the interior of the ventricles, is by no means so very evident a disease. It may, and often does, pass to a fatal termination, without other symptoms than mere coma and stupor. These symptoms were present to a very slight degree at the commencement of this case, and were regarded as probable evidence of arachnitis, not of the intense form which it afterwards assumed, but of that mild sub-acute variety, which is so frequent in cases of pneumonia.

Even in the last stage of the disease, although the symptoms became very evident, there was no reason for believing that the lesion of the brain was nearly as considerable as was afterwards found.

In the present instance the inflammation of the brain was the more obscure, because it occurred during the course of pneumonia: the primary disease is much less apt to be latent.

The lesion of the lung corresponded accurately with the extent and period of the disease, as ascertained by the physical signs and the expectoration. It was confined to the right lung, but had reached the period at which the disease is generally most fatal; that is, the passage from the second into the third stage.

I did not intend to allude to this case as an illustration of the complication of endo-carditis. The affection of the brain was so much more prominent, and exercised so much more evident an influence upon the fatal termination than the cardiac inflammation, that I had reference in relating the case, only to the cerebral complication. During life, the attention was rather directed to the lungs and brain than to the heart, but I observed that the pulsations were confused and irregular. On examination after death, we detected a very evident endo-carditis, which had produced ulceration of the mitral valve, and a partial erosion or ulceration of the semi-lunar valves of the aorta. Still there was no thickening, to a sufficient degree to impede the action of the valves, or to give rise to either an intense blowing or rasping sound. The usual signs of endo-carditis were much more obscure than they are in ordinary cases of the disease; this was the result of that general law of pathology to which I have so often referred. That is, secondary inflammations are much less evident than the primary affections of the same organs.

